# Gut Microbial-Derived Short Chain Fatty Acids: Impact on Adipose Tissue Physiology

**DOI:** 10.3390/nu15020272

**Published:** 2023-01-05

**Authors:** Karolline S. May, Laura J. den Hartigh

**Affiliations:** 1 Department of Medicine, Division of Metabolism, Endocrinology, and Nutrition, University of Washington, Seattle, WA 98109, USA; 2 UW Medicine Diabetes Institute, 750 Republican Street, Box 358062, Seattle, WA 98109, USA

**Keywords:** gut microbiome, short-chain fatty acids, adipose tissue, obesity, inflammation, acetate, propionate, butyrate, metabolism

## Abstract

Obesity is a global public health issue and major risk factor for pathological conditions, including type 2 diabetes, dyslipidemia, coronary artery disease, hepatic steatosis, and certain types of cancer. These metabolic complications result from a combination of genetics and environmental influences, thus contributing to impact whole-body homeostasis. Mechanistic animal and human studies have indicated that an altered gut microbiota can mediate the development of obesity, leading to inflammation beyond the intestine. Moreover, prior research suggests an interaction between gut microbiota and peripheral organs such as adipose tissue via different signaling pathways; yet, to what degree and in exactly what ways this inter-organ crosstalk modulates obesity remains elusive. This review emphasizes the influence of circulating gut-derived short chain fatty acids (SCFAs) i.e., acetate, propionate, and butyrate, on adipose tissue metabolism in the scope of obesity, with an emphasis on adipocyte physiology in vitro and in vivo. Furthermore, we discuss some of the well-established mechanisms via which microbial SCFAs exert a role as a prominent host energy source, hence regulating overall energy balance and health. Collectively, exploring the mechanisms via which SCFAs impact adipose tissue metabolism appears to be a promising avenue to improve metabolic conditions related to obesity.

## 1. Introduction

### 1.1. Pathophysiology of Obesity

The prevalence of obesity has escalated in recent years, thus representing a serious impediment to public health worldwide [1]. Recent trends in the United States estimate that over 40% of the US population has either overweight or obesity [2], with this rate likely to be even higher among adults by 2030 [3,4]. Additionally, epidemiologic studies indicate that obesity-related pathological conditions, including type 2 diabetes (T2D) [5], dyslipidemia [6], coronary artery disease (CAD) [7,8], hepatic steatosis [9], and certain cancers [10,11,12], will continue to increase, potentially reducing life expectancy and increasing healthcare costs [13,14]. In this regard, there is an urgent need to identify biological mechanisms and effective approaches to manage obesity and its risk factors.

Obesity is a multifactorial disorder characterized by a sustained energy imbalance (energy intake > energy expenditure) [15], leading to adverse physiological consequences across individuals [1,16]. In obesity, adipose tissue (AT) metabolism plays an important role in driving the negative effects of increased adiposity [16,17]. As such, marked nuances in AT distribution throughout the body as well as AT phenotypes (white, brown, and beige) [18] play a key role in various metabolic complications [16]. Moreover, due to the energy imbalance that characterizes obesity, white adipose tissue (WAT) undergoes expansion and remodeling coupled with changes in AT-resident immune cells [19,20,21]. Failure to modulate WAT mass via hypertrophy in adipocytes, hyperplasia of resident pre-adipocytes, or combined mechanisms [18] drives excessive ectopic fat deposition in peripheral tissues (i.e., heart, liver, skeletal muscles, pancreas), imbalanced energy storage, increased reactive oxygen species (ROS) production, inadequate vascularization, hypoxia, hormonal dysregulation, and low-grade systemic inflammation, with macrophage infiltration and polarization [17,21,22,23,24,25]. Hence, through interactions with distinct organs and a multitude of signaling pathways, WAT contributes to cardiometabolic health, ultimately impacting whole-body energy homeostasis [17,21,26]. A more detailed description of the metabolic changes in AT elicited by obesity will be further discussed herein. Given the overall health burden, various hypotheses have emerged to explain the underlying causes of obesity that are summarized in [27,28,29], but will not be the main focus of this review.

### 1.2. Obesity and the Gut Microbiota

**Figure 1:**
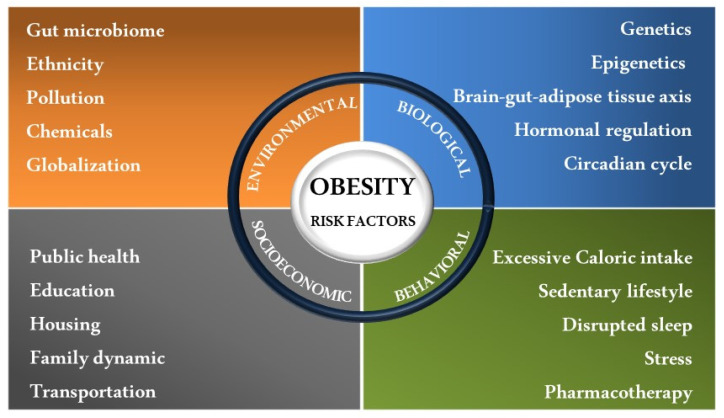
**Obesity-attributable risk factors.** Targetable individual behavioral, societal, biological, and environmental factors that contribute to the complex etiology of obesity and obesity-related metabolic complications in humans.

Evidence has suggested that the etiology of obesity is quite complex and depends on several elements [1,29,30]. A combination of individual behaviors and societal, biological, and environmental factors may affect body weight and adiposity [1,29,30] and should therefore be utilized to inform therapeutic approaches to obesity management and treatment. Among the environment-attributable factors related to obesity, research in animal models [31,32,33,34,35] and humans [36,37,38] has shown that the gut microbiota is a new keystone linked to obesity-related metabolic complications [39,40]. Previous research highlights the relevant contribution of the intestinal microbial ecosystem to energy harvest from the diet as well as its impact on genetic and diet-induced adiposity [41,42,43,44,45], suggesting that targeting the gut microbiota may be an avenue to treat environment-related obesity and other related metabolic diseases. Figure 1 depicts the wide spectrum of factors that influence obesity and related comorbidities.

The gut microbiota harbors a wide variety of bacterial genera, the majority of which are represented by the phyla *Firmicutes* and *Bacteroidetes*. These can be further distributed across many diverse bacterial species [46,47,48], thereby conferring the host with an individualized microbiota composition [46,49,50]. Interestingly, differences in the composition and distribution of the microbiota have been correlated to adiposity, as revealed by mechanistic studies [35,37]. The composition of the diet, how nutrients are ingested, intestinal transit time, and the duration of fasting throughout the day can greatly affect microbial populations, which could modulate energy balance and body weight [51,52]. Initially, Ley et al. showed that obesity correlates with a decreased abundance of *Bacteroidetes* with a higher proportion of *Firmicutes*, whereas lean individuals harbored a higher proportion of *Bacteroidetes* by a corresponding degree [35]. Such profound changes in the bacterial community structure, associated with an alteration in body weight, were observed regardless of food consumption in genetically obese mice [35]. In parallel, reduced levels of *Bacteroidetes* were found in cecal samples from leptin-deficient (*ob*/*ob*) obese mice relative to their lean counterparts [32], an effect also observed in obese humans [37]. However, an accumulation of subsequent metagenomics data revealed that the *Firmicutes* to *Bacteroidetes* relative ratio is not a consistent indicator of obesity status, suggesting far more complexity regarding the imbalance of gut microbial phyla related to metabolic disease status [53,54,55,56]. The notion that alterations in microbial ecology can modulate obesity, or at least can contribute to a host-mediated adaptive response to energy imbalance, has led to many studies in germ-free (GF) and conventionalized mice [31,32,33,34,57]. Proof-of-concept experiments showed that an “obese microbiome” was capable of driving host adiposity through increased adipocyte lipoprotein lipase (LPL) activity, adipocyte hypertrophy [31], reduced proportions of *Bacteroidetes*, and an enrichment in genes related to energy metabolism pathways in adult GF mice [32,34]. These findings suggest that the gut microbiota may efficiently harvest energy from a given diet and act as a crucial environmental factor in regulating energy harvesting, storage, and expenditure [31,32,34]. Other mechanisms that explain how gut dysbiosis mediates obesity pathophysiology include increased intestinal permeability accompanied with metabolic endotoxemia, dysregulated gut immunity, contribution to low-grade systemic inflammation, and hormonal imbalance in the gut–brain–adipose axis [58,59,60,61,62]. Furthermore, particular gut microbial metabolites such as short-chain fatty acids (SCFAs), the majority of which include acetate, propionate, and butyrate, could also contribute to host energy homeostasis and have been extensively investigated in this context [63,64,65,66,67,68,69,70,71].

While gut microbiota are intimately involved in host energy metabolism, their contribution to weight loss, and potential direct signaling to adipose tissue, is less well-defined. Herein, the focus of this review will be on the direct influence of circulating gut-derived SCFAs on adipocyte metabolism in vitro. We also will discuss potential mechanisms whereby microbial SCFAs impact adipose tissue and adipocyte metabolism in vivo, hence regulating overall energy balance and health. Literature that contributed to this review was found using Pubmed, with search terms that included combinations of the terms SCFAs, adipose tissue, and obesity in vitro and in vivo. Collectively, exploring SCFAs as potential targets that impact the gut–adipose tissue axis may be a promising avenue to improve metabolic conditions related to obesity.

## 2. Gut Microbial-Derived Short Chain Fatty Acids (SCFAs) and Obesity

The composition and functional properties of the gut microbiota affect whole-body metabolism, thus playing a role in obesity and its complications [72,73]. As such, evidence indicates that nutritional strategies, including consumption of a fiber-rich or plant-based diet, seem to improve host bacterial richness and overall colonic health, with the potential to mitigate the metabolic complications of obesity [74,75,76,77]. Particular microbial metabolites, including SCFAs derived from the fermentation of indigestible dietary fibers [78,79], exert beneficial effects on both the host and its microbiome. SCFAs serve as (1) an energy source for both microbiota and the host to maximize gut health and (2) circulating factors facilitating the crosstalk between the host and gut microbiota [72,74]. Indeed, adipose tissue boasts a relatively high expression of SCFA receptors relative to other tissues (described in more detail in Section 3). In this context, precise roles of SCFAs and their direct impact on host adipose tissue metabolism have been underexplored [74,78,79].

SCFAs refer to organic monocarboxylic acids with fewer than six carbons, encompassing primarily acetate (C2), propionate (C3), and butyrate (C4) at an approximate molar ratio of 3:1:1, respectively, in the intestinal lumen [78,80]. These small molecules account for approximately 95% of all SCFAs and are produced by specific bacterial strains depending on the type and amount of fiber consumed, the extent of substrate degradation, and colonic transit time [81,82,83]. Acetate, the most abundant systemic SCFA, and propionate are primarily produced by particular strains of *Bacteroidetes* (i.e., *Bacteroides* spp., *Bifidobacterium* spp., *Lactobacillus* spp.), whereas butyrate-producing bacteria are primarily of the phylum *Firmicutes* (i.e., *Enterococcus* spp., *Eubacterium* spp., *Roseburia* spp.) [84,85,86]. SCFAs circulate at high levels in rodents and humans, typically in the range of several hundred micromolar. In contrast to what is observed in the intestinal lumen, the majority of circulating SCFAs in mice and humans are represented by acetate, with a smaller proportion represented by propionate and butyrate [87,88,89], suggesting a potentially important systemic function of these SCFAs.

**Figure 2:**
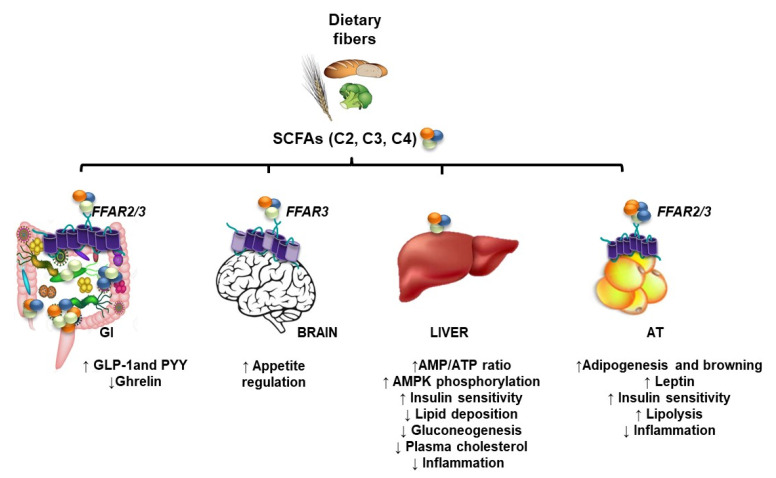
**Overview of host responses to short chain fatty acids (SCFAs).** SCFAs acetate (C2), propionate (C3), and butyrate (C4) derive from the microbial fermentation of non-digestible dietary fibers. These metabolites are utilized by the host, thus promoting a range of beneficial metabolic responses in peripheral tissues beyond the gut. After being absorbed through simple diffusion or signaling-mediated free fatty acid receptors (FFAR2 or FFAR3), SCFAs stimulate hormonal release (GLP-1, PYY, Ghrelin) in the gastrointestinal tract (GI), which directly signal the brain to monitor energy intake and appetite regulation. At micromolar concentrations, SCFAs enter the circulation and reach the liver via the portal vein to attenuate glucose production and lipid deposition and inflammation, possibly by activating AMPK. Additionally, SCFAs may directly or indirectly affect the brain and adipose tissue (AT) and, therefore, modulate lipid buffering capacity, adipokine secretion, insulin sensitivity, and inflammation.

Dietary substrates are fermented to SCFAs by gut microbes, then efficiently absorbed via either simple passive diffusion or anion sodium/potassium (NA^+^/K^+^) exchange in the cecum and proximal colon [80,90]. Once absorbed by colonocytes, SCFAs are highly metabolized in the colonic epithelium, which preferentially uses butyrate, followed by acetate, as the main energy sources under physiological conditions [91,92]. Alternatively, residual butyrate and propionate as well as up to 70% of acetate are metabolized by hepatocytes for gluconeogenesis. Finally, the remaining acetate is oxidized by muscle cells for energy production [93,94]. A study conducted by Cummings et al. demonstrated that SCFAs are present in the portal vein and the liver at high concentrations, then progressively decline in the peripheral systemic circulation, but remain in the range of several hundred micromolar systemically [78]. In addition to stool, some excretion of SCFAs from the body may occur in urine and breath (~5 to 20%) [95]. While SCFA have a clear positive impact on the intestinal environment via reduction of colonic pH [96] and modulation of microbial and colonic energy homeostasis [74,97], SCFAs counteract obesity-induced inflammation [98] and regulate glucose [99] and lipid metabolism [100] (Figure 2). To what extent these effects directly or indirectly control the physiological function of host adipose tissue will be discussed in further sections.

### 2.1. Gut Microbial-Derived SCFAs in Mice with Obesity

Several studies in mice point towards a correlative link between body weight and gut-derived SCFA levels. In a genetic mouse model of spontaneous obesity and diabetes, analysis of the gut microbiome and SCFA profiles showed that the abundance of *Bacteroidetes* decreased in parallel with plasma acetate levels [101]. In the same mice, the relative abundance of *Firmicutes* and fecal butyrate were higher relative to their littermate controls, suggesting that shifts in gut microbiota may lead to the loss of type and quantity of SCFAs [101]. In contrast to genetically-induced obesity, nutritionally obese rodents fed a hypercaloric diet had a lower abundance of *Bacteroidetes* as well as a reduction in fecal acetate and propionate content over time [102], an effect that was reversed by the addition of dietary fiber [103]. Another study also showed that shifting the gut microbial composition towards higher SCFA production using dietary fiber rescued the obesity-inducing effects of a high fat diet in mice [104], suggesting that dietary strategies to increase endogenous SCFA production could mitigate obesity.

Our recent findings indicate that an obesogenic diet followed by distinct weight loss interventions alter gut microbial structure and composition, which also impacted SCFA production in mice [88]. In this study, low-density lipoprotein receptor (LDLR) knockout mice administered a high fat high sucrose diet (HFHS) supplemented with *trans-10*,*cis-12* conjugated linoleic acid (*t10*,c12 CLA, a dietary compound that promotes weight loss) presented an enrichment in butyrate-producing bacterial species as well as an elevated fecal butyrate content in conjunction with weight loss and decreased adiposity [88]. Such weight loss and gut microbiota changes were also associated with increased systemic acetate. Collectively, previous work suggests that changes in body weight are coincident with changes in fecal and systemic SCFAs in mice, but whether such changes are the direct result or causal of weight loss remains unknown. Additional studies on the associations between fecal SCFA and obesity in animal models have been reviewed by us previously [105].

### 2.2. Gut Microbial-Derived SCFAs in Humans with Obesity

In contrast with animal models, associations between body weight and SCFAs in humans are less clear. While the administration of probiotics has been shown to associate with reductions in body weight and adiposity in a recent meta-analysis in humans [106], whether these beneficial effects of probiotics involved SCFAs was not assessed. To complicate the matter, fecal SCFA levels have been reported to be lower in children with obesity [107], higher in pediatric subjects with obesity [108], elevated in adults with obesity relative to lean healthy control subjects [109,110], and to decrease with age [111], observations that are quite contradictory. A recent meta-analysis has shown that among seven studies evaluated, people with obesity had a significantly higher fecal SCFA content compared to lean controls [112]. Another study similarly showed that among 490 Chinese adults aged 30–68, plasma SCFA levels positively associated with body mass index (BMI) [113]. An investigation led by Schwiertz et al. observed that the ratio of *Firmicutes* to *Bacteroidetes* shifted in favor of *Bacteroidetes* in overweight and obese humans, with a significant increase in fecal propionate [55]. Other studies suggest that fecal SCFAs are negatively associated with BMI, waist circumference, and visceral adiposity in humans [114,115,116]. Thus, whether SCFAs are involved in energy balance in humans is unclear and requires further study.

## 3. SCFA Receptor Signaling

**Figure 3:**
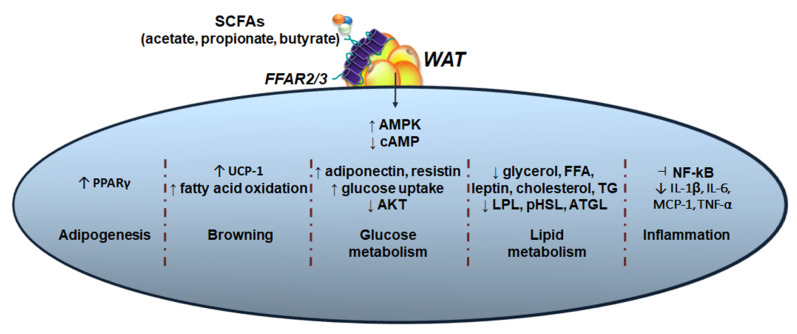
**SCFA-FFAR2/3 signaling pathways in adipose tissue**. Gut bacterial SCFA-activated free fatty acid receptors 2 and 3 (FFAR2/3) modulate host adipose tissue physiology, thus regulating body weight and whole-body metabolic homeostasis. In white adipose tissue (WAT), studies indicate that SCFA-FFAR2/3 putatively impact nutritional status and energy balance through signaling via various molecular pathways including adipogenesis [135,136,137,138,139,140,141], browning [133,135,142], glucose [132,142,143] and lipid metabolism [131,132,133,138,139,142,144,145], and inflammation [132,133,135,137,140,142,146,147,148].

Microbial-derived SCFAs are absorbed into the bloodstream and impact whole-body physiology through mechanisms that may involve G protein-coupled receptors (GPCRs), which are also termed free fatty acid receptors (FFARs) [117,118]. FFAR2 (GPR43) and FFAR3 (GPR41) are selectively activated by SCFAs, thus activating metabolic pathways in the setting of obesity and obesity-related diseases [119,120]. Acetate preferably binds to FFAR2, whereas propionate and butyrate show high affinity to FFAR3 as revealed in HEK293 cells [121]. FFAR2/3 have been extensively described as ~40% homologous to each other in humans and mice, with proximity on chromosomes 1 (humans) and 7 (mice) [117,121]. In terms of specificity, FFAR2 displays a dual coupling through G_i/o_ and pertussis toxin-insensitive G_q_ protein, further influencing cyclic adenosine monophosphate (cAMP), the extracellular signal-regulated protein kinase 1/2 (ERK1/2) cascade, and calcium channels, whereas FFAR3 couples exclusively with the G_i/o_ protein family [120,121]. Both cell surface receptors are detected in a variety of organs beyond the intestine [122,123,124,125], with expression described in immune cells [126,127,128], liver [129,130], and adipose tissue depots [131,132,133]. The importance of the hydroxycarboxylic acid receptor HCA2 (GPR109A) and olfactory receptor 78 (OLFR78) as potential SCFA receptors has been less extensively explored [133,134]. In this section, we will discuss the putative effects of SCFAs on host adipose tissue that likely occur via activation of FFAR2/3 (Figure 3) and whether these receptors play a role as therapeutic targets for metabolic conditions.

### 3.1. FFAR2 and FFAR3 Signaling in Adipose Tissue of Rodents

FFAR2 and FFAR3 are expressed in nearly all metabolically active tissues, including adipose tissue [117,120,149], thus promoting a potential association between the gut microbiota, SCFAs, and adipose tissue. Notably, given that FFAR2/3 share an overlapping range of ligands [149], it is possible that their metabolic roles in adipose tissue are partially redundant, leading to the potential for conflicting data in this area. The majority of studies on the function of FFAR2/3 activation in cultured adipocytes, rodent models, and human studies demonstrate that SCFA-activated FFAR2/3 impact body weight and fat storage capacity in adipocytes [133,135], as well as other adipose tissue functions such as glucose and lipid homeostasis [132,137], adipogenesis [136,137], lipolysis [138,139,140], adipokine release [131,144,150], and low-grade inflammation [146,147,148].

Several studies have reported that SCFAs impart metabolic benefits on host energy homeostasis, which is likely mediated by FFAR2 and/or FFAR3 [132,133,135,142]. A study conducted by Lu et al. showed that a 5% (wt/wt) mixture of SCFAs incorporated into a high fat diet (HFD) reduced body weight, total cholesterol, and inflammatory mediators such as IL-1ꞵ, IL-6, and monocyte chemoattractant protein (MCP-1), potentially via increased expression of FFAR2/3 in the epididymal adipose tissue in mice [133]. SCFA-mediated increases in FFAR2/3 mRNA levels were also accompanied by elevated expression of the insulin-sensitizing adipokine adiponectin, increased expression of fat oxidation genes, and decreased markers of lipolysis [133], suggesting beneficial metabolic effects via adipose tissue. To a similar extent, FFAR2/3 activation positively correlated with adipogenesis and thermogenesis [133], reinforcing the beneficial roles of these receptors in energy expenditure and obesity. In addition, a study in which mice were fed a HFD with and without the addition of individual SCFA for 5 weeks showed that SCFAs reduced weight gain and visceral fat mass, at least partly by reducing energy intake, and contributed to changes in plasma cholesterol, free fatty acids (FFA), and glucose [135]. In addition to potentially modulating lipid and glucose homeostasis via FFAR2/3 mRNA upregulation, these data revealed that SCFAs attenuate the inflammatory markers IL-1ꞵ and IL-6 while improving insulin sensitivity, hydrolysis of triglycerides, fatty acid oxidation, WAT browning, adipogenesis, and mitochondrial function in WAT [135].

SCFA-mediated FFAR2/3 signaling plays crucial roles in glucose and lipid metabolic pathways [142], and a few studies showcasing perturbed FFAR2 expression in mice underscore the important association between SCFA and cardiometabolic risk in models of obesity. Bjursell et al. generated the first mice globally deficient in FFAR2 (FFAR2-KO). When fed a HFD for 35 weeks, FFAR2-KO mice exhibited lower body fat mass and retroperitoneal WAT despite increased food intake, which was likely offset by increased energy expenditure, elevated core body temperature, and a reduced respiratory exchange ratio (RER), indicating increased fat oxidation [142]. Adipose tissue homeostasis was also improved, as mirrored by reduced macrophage infiltration and elevated adiponectin production. Importantly, FFAR3 expression was increased in WAT in FFAR2-KO mice fed a chow diet, a potential compensation for the loss of FFAR2. Moreover, HFD-fed FFAR2-KO mice had higher brown adipose tissue (BAT) activity, without significant alterations in genes involved in thermogenesis [142]. These findings suggest that this first FFAR2-deficient model improved obesity phenotypes [142]. In stark contrast, a second FFAR2-KO mouse model later reported by Kimura et al. showed that mice globally deficient in FFAR2 fed either normal chow or a HFD exhibited increased body weight and adiposity due to decreased energy expenditure, a phenotype that was dependent on an intact microbiota [132]. Further, transgenic mice with overexpression of FFAR2 specifically from adipocytes were protected against HFD-induced obesity and insulin resistance [132]. Collectively, the two mouse models reporting genetic perturbations in FFAR2 have yielded completely opposing phenotypes, requiring further study. Additional studies on FFAR2/3-mediated responses to SCFAs should be further explored in animal models and in human adipose tissue, which could support the design of dietary interventions and particular strategies to increase systemic availability of SCFAs. Table 1 lists studies describing mechanistic changes in FFAR2/3 expression levels in adipose tissue in rodents, highlighting the potential for FFAR isoforms as possible targets to improve diet-induced obesity, long-term energy balance, and weight management.

Several groups have also examined phenotypes related to body weight and/or obesity in FFAR3-KO mice. Jeffrey Gordon’s group described the first global FFAR3-KO mice in 2008 [151]. FFAR3-KO mice were leaner than their WT counterparts in the presence of an intact microbiota on a chow diet [151]. By contrast, when fed a high fat diet, FFAR3-KO mice exhibited increased body weight and adiposity with reduced energy expenditure [152,153], suggesting that HFD feeding is required for FFAR3-mediated effects on body weight. However, an additional FFAR3-KO mouse model showed that HFD-induced obesity was reduced compared to WT controls, potentially due to increased energy expenditure [142]. Thus, again, there is little consensus regarding the impact of FFAR2/3 on body weight and adiposity.

Beyond the scope of the current review, SCFA-activated FFAR2/3 can modulate energy homeostasis and indirectly affect host adipose tissue by increasing gut hormonal release and triggering inflammatory responses [125,151,154,155]. Global FFAR3-KO mice fed a carbohydrate-rich diet exhibited a decrease in leptin secretion as well as the enteroendocrine cell-derived peptide YY (PYY), a hormone that selectively reduces gut motility and impacts energy harvesting from the diet, thus implying that FFAR3 may be required for PYY-mediated effects [151]. These results were confirmed by Tolhurst et al., in which mice deficient in FFAR2 or FFAR3 exhibited lower secretion of PYY and the incretin glucagon-like peptide 1 (GLP-1) followed by a disruption of glucose tolerance [154]. In parallel, SCFA-mediated gut hormone secretion was also impaired in FFAR2/3^-/-^ colonic cultures in vitro [154]. Conversely, a mechanistic study showed that butyrate and propionate protect against diet-induced obesity and regulate glucose-dependent insulinotropic peptide (GIP) and GLP-1 secretion independently of FFAR3, indicating a minor role of this FFA receptor in host metabolism [155]. In this regard, inflammation by SFCA-activated FFAR2/3 was assessed in mice fed SCFAs or given injections of antibodies that delay the immune response. The data showed that mice lacking FFAR2/3 did not exhibit major histological changes or evidence of altered inflammatory mediators in the intestine, which can be related to defective SCFA signaling. In sum, the authors emphasize the importance of FFAR2/3 to mitigate further whole-body inflammation and associated complications [155].

**Table 1:** Short chain fatty acid receptor signaling in host adipose tissue metabolism.

Model	Experimental Design	FFAR Expression	Metabolic Response	Reference
C57BL6/J mice	HFD-SCFA	↑ FFAR2/3	↓ body weight, total cholesterol	Lu et al., 2016 [133]
	mixture: 5% (wt/wt^−1^) acetate, propionate, and butyrate for 16 weeks		↓ IL-1β, IL-6, MCP-1	
			↑ adiponectin, resistin	
			↑ lipolysis and FFA oxidation	
			↑ adipogenesis and mitochondrial biogenesis	
C57BL6/J mice	HFD-SCFA individualized: 5% (wt/wt^−1^) acetate, butyrate, or butyrate for 5 weeks	↑ FFAR2/3	↓ body weight, total cholesterol, FFA and glucose.	Jiao et al., 2021 [135]
			↓ energy intake	
			↓ IL-1β, IL-6	
			↑ insulin sensitivity	
			↑ lipolysis and FFA oxidation	
			↑ browning, adipogenesis and mitochondrial function	
FFAR2-KO mice	HFD for 35 weeks	↔ FFAR2 ↑ FFAR3	↓ body weight, retroperitoneal WAT	Bjursel et al., 2011 [142]
			↑ energy intake/expenditure, core body temperature, RER	
			↓ inflammation	
			↑ adiponectin	
			↑ BAT activity	
			↑ insulin sensitivity	
FFAR2-KO	HFD for 12 weeks	↑ FFAR2	FFAR2-KO mice: ↑ body weight	Kimura et al., 2013 [132]
			↓ insulin sensitivity	
			↑ inflammation, TNF-α	
aP2-FFAR2tg			aP2-FFAR2tg: ↓ body weight	
			↑ insulin sensitivity	
			↓ inflammation, TNF-α	
			↓ LPL activity	

### 3.2. FFAR2 and FFAR3 Signaling in Adipocytes In Vitro

Several in vitro studies have contributed to what we know regarding the influence of SCFA-activated FFAR2/3 on adipocyte metabolic regulation, informing the roles of SCFAs in the onset and progression of obesity [136,137,138,139,140,143,145]. Hong et al. demonstrated in 3T3-L1 adipocytes that microbial-derived acetate and propionate promote adipogenesis by increasing peroxisome proliferator-activated receptor gamma (PPARγ) expression and lipid accumulation and blunt lipolytic activity, mainly through FFAR2 [139]. Similar findings were confirmed years later by another group that showed that 3T3-L1 adipocytes exposed to acetate and propionate for 4 h exhibited increased FFAR2 expression with decreased evidence of lipolysis, namely, reduced free fatty acids and glycerol release [138]. In line with this, supra-physiological concentrations of acetate target FFAR2 via G_i_/_o_, thus suppressing insulin-induced protein kinase B (AKT) phosphorylation and LPL activity as well as fatty acid uptake [132]. Quantitative and qualitative assays determined that FFAR2, but not FFAR3, is expressed at late stages of adipocyte differentiation and impacts intracellular lipid content [144]. At micromolar doses, propionate did not influence lipid accumulation or leptin secretion in 3T3-L1 adipocytes, indicating that earlier expression of FFAR2 might be required to modulate such adipogenesis markers [144]. Additional studies have yielded mixed results [131,143]. First, in fully differentiated 3T3-L1 adipocytes, an exposure to distinct concentrations of propionate significantly increased insulin-stimulated glucose uptake, at least in part, through FFAR3. Moreover, silencing FFAR3 using small interfering RNA (siRNA) blunted approximately 30% of the SCFA-induced glucose effects in vitro, implying a potential role for FFAR3 to modulate glucose uptake in adipocytes [143]. Second, Xiong et al. examined whether SCFAs are required to stimulate leptin release via FFAR3 in mouse primary adipocytes. The results indicated that SCFA-upregulated FFAR3 led to leptin secretion, an effect that was abolished via pertussis toxin treatment. In addition, data showed that a marked decrease in cAMP levels supports the exclusive G_i_-coupled pathway mediated by FFAR3 [131]. The roles of FFAR3 in leptin stimulation of adipocytes in response to SCFAs was then explored in adipocytes from FFAR3-KO mice [144]. Interestingly, the authors concluded that FFAR3 was undetectable at a molecular level in mouse adipose tissue, in contrast to previous reports [131,143]. Additionally, reduced leptin production and lipolysis, in the presence of SCFAs, might be related to the downregulation of FFAR2 rather than other unknown mechanisms, suggesting a crucial interplay between the FFA receptors [144].

Despite accumulating evidence to support the influence of microbial-derived SCFA activation of FFAR2/3 in the regulation of host adipose tissue biology and physiology, studies in humans are needed to understand such ambiguous findings. Al-Lahham et al. demonstrated for the first time that propionate counteracts inflammation and increases leptin secretion via omental (OAT) and subcutaneous adipose tissue (SAT) explants from female subjects [150]. Such metabolic responses were attributed to high expression of FFAR2/3, confirmed by blocking the G_i/o_ pathway with pertussis toxin [150]. Next, preadipocytes isolated from human OAT were incubated with acetate and propionate to evaluate the effects of FFAR2 agonists on adipogenesis [137]. Unlike what has been described in murine studies, results from Dewful et al. suggested that FFAR2 is not implicated in adipocyte differentiation, as exemplified by adipocyte fatty acid-binding protein 2 (aP2) expression, but might be associated with inflammatory outcomes in humans [137]. Moreover, propionate inhibited lipid accumulation and adipogenesis in human adipose-derived mesenchymal stem cells (MSCs) via FFAR2 [136]. Therefore, the role of FFAR2 protein should be further elucidated via the use of knockdown and overexpression in human adipocyte models.

To assess the differential effects of SCFAs on targeting FFA receptors, Jocken et al. proposed that individual SCFAs of a mixture could alter adipocyte lipolysis, thus impacting whole-body energy homeostasis [145]. In human multipotent adipose tissue-derived stem (hMADS) cells, which resemble human white adipocytes, high doses of acetate conferred an anti-lipolytic response followed by a reduced phosphorylation of hormone sensitive lipase (HSL) at serine 650 (pHSL_SER650_) in a FFAR2/3-dependent manner [145]. Collectively, the apparent discrepancies among these in vitro and ex vivo findings may stem from differences in SCFA concentrations and length of SCFA exposure, mouse strains with different backgrounds, age, sex, the use of human adipose tissue harvested from distinct sites, and varied metabolic conditions employed in each study protocol. In Table 2 we describe findings from experimental analyses in vitro and ex vivo, including conflicting metabolic outcomes.

## 4. SCFA Potential for Targeting Histone Deacetylases

The majority of findings to date indicate that activation of FFAR2/3 by SCFAs could be a promising anti-obesity strategy, with direct implications for adipose tissue function. Moreover, epigenetic mechanisms shed light on how SCFAs promote beneficial rather than detrimental metabolic responses, thus impacting whole-body energy homeostasis [156,157,158]. SCFAs, mainly butyrate, act as natural histone deacetylase (HDAC) inhibitors to epigenetically regulate chromatin acetylation and gene transcription involved in cell proliferation, differentiation, apoptosis, fatty acid oxidation, and inflammation [159,160,161,162,163]. Of note, SCFA-mediated HDAC inhibitory activity depends on the concentration of SCFAs and on which cell type or tissue the gut metabolites may target [159]. Li et al. showed that butyrate contributes to the differentiation of pre-adipocytes into mature adipocytes via increased histone acetylation at the promoters of PPARγ and CCAAT enhancer-binding protein alpha (CEBPα), leading to upregulation of these adipogenic genes [164]. Moreover, additional work confirmed that butyrate and propionate, but not acetate, impact the rate of lipolysis via their shared activity as HDAC inhibitors in 3T3-L1 adipocytes, thus suggesting a possible molecular pathway to mitigate obesity-related outcomes [165]. As pointed out by the authors, in vivo analyses are required to confirm in detail whether such HDAC inhibitors can differentially impact whole-body metabolism [164,165]. In line with that, a study in type 2 diabetic rats fed a HFD revealed that butyrate treatment reduced body weight and adiposity while improving glucose homeostasis through hyperacetylation of histone H3, thus likely inhibiting HDAC activity as neither FFAR2 nor FFAR3 were modulated [162]. Similarly, butyrate activates the phosphorylation of the mitogen-activated protein kinase 3/p38/p38-regulated activated protein kinase (MKK3/p38/PRAK) signaling pathway and protects against HFD-induced cardiometabolic disruptions in response to HDAC inhibition in diabetic and obese mice [163]. Moreover, a short-term intervention with butyrate counteracted HFD-induced adiposity and insulin resistance, while promoting histone acetylation on the promoters of genes encoding adiponectin receptors and uncoupling proteins in C57BL6/J mice [161]. Lastly, adipose tissue levels of HDAC were significantly decreased in obese rats given acetate, with subsequent normalization of metabolic homeostasis via modulation of PPARγ and suppression of oxidative stress [166]. In summary, in addition to the possibility of therapeutically targeting HDAC, to what extent SCFAs selectively inhibit HDAC requires further investigation as another potential mechanism to manage obesity.

## 5. Gut Microbial-Derived SCFAs Influence Host Adipose Tissue Inflammation

Adipose tissue is a dynamic tissue that contributes to local and systemic low-grade inflammation, thus orchestrating the progression of insulin resistance and pathophysiology of obesity [18,23]. Hence, chronic inflammation in adipose tissue promotes the recruitment and infiltration of various immune cells, including macrophages and T cells, which regulate the secretion of cytokines and activation of inflammatory signaling cascades [25,26]. While the contribution of adipose tissue inflammation to the pathology of obesity has been established, it is worth discussing this paradigm in the context of microbial-derived SCFAs in vitro and in vivo [140,147,167,168,169,170,171].

Effects of SCFA on inflammatory responses in monocytes and macrophages have yielded conflicting results. A study conducted by Al-Lahham et al. reported that propionate impacts human omental OAT and THP-1-derived macrophages, resulting in down-regulation of cytokines and chemokines, crucial mediators linked to ATM activation [147]. Indeed, the expression of specific macrophage markers was impacted by propionate, suggesting that macrophages could be targets for SCFAs within adipose tissue, either directly or indirectly [147]. In a different study, co-stimulation with SCFAs and the pro-inflammatory mediators tumor necrosis factor alpha (TNF-α) and lipopolysaccharide (LPS) exerted opposing effects in monocytes and macrophages. Acetate, in combination with TNF-α, increased the expression of chemokines in THP-1 monocytic cells, but decreased MCP-1 in macrophages, implying a paradoxical SCFA-mediated inflammatory response that is dependent on cell type, culture conditions, and SCFAs concentration [171]. Immunomodulatory effects of SCFAs were further explored by Ohira et al. [140,167]. The authors showed that butyrate blunts inflammatory responses and activation of cell signaling pathways coordinated via the interaction of 3T3-L1 adipocytes and RAW264.7 macrophages [140,167]. In a similar co-culture experimental design, butyrate increased prostaglandin E2 (PGE2) production, which is correlated to adiposity, and signaling factors including cyclooxygenase-2 (COX2) and cytosolic phospholipase A2 (cPLA2), in a dose-dependent manner [167]. Thus, whether SCFAs promote or reduce inflammatory responses in monocytes and macrophages remains to be determined.

Importantly, ATM, upon stimulation with local environmental factors, may be further divided into “classically activated” (M1), “alternatively activated” (M2), or “metabolically activated” (MMe) macrophage phenotypes [25,172]. Bone marrow-derived macrophages exposed to IL-4 and different concentrations of butyrate activated the histone H3 lysine 9/signal transducer and activator of transcription 6 (H3K9/STAT6) signaling pathway and enhanced M2-related gene expression and chemokines, indicating a putative role of such SCFAs to facilitate M2 macrophage polarization [170]. The effects of butyrate inducing the M2 macrophage profile may contribute to adipose tissue homeostasis, thus alleviating obesity and related comorbidities. In a diet-induced obesity mouse model, butyrate increased M2 relative to M1 macrophages and regulatory T cells and associated genes, whereas pro-inflammatory markers were reduced in white adipose tissue [169]. Furthermore, analysis in 3T3-L1 adipocytes showed that butyrate markedly decreased the expression of endoplasmic reticulum (ER) stress proteins, suggesting a potential to mitigate inflammation and cell autophagy [169]. Recent work in peripheral blood monocytes and ATM from obese individuals provided evidence that SCFAs impact molecular pathways that reduce systemic inflammation, evidenced by decreased nuclear factor kappa B (NF-ᴋB), mitogen-activated protein kinases (MAPK), and pro-inflammatory cytokines following weight loss [168]. A list of relevant studies describing the modulation of adipose tissue inflammation by SCFAs are listed in Table 3 and additionally reviewed in [105].

## 6. Administration of SCFAs to Impact Adipose Tissue

Proof-of-concept studies have investigated whether SCFAs influence adipose tissue and whether they ultimately impact energy balance and overall metabolism in rodent models and humans [68,71,133,173,174,175,176,177,178,179,180,181,182,183,184,185,186,187,188]. In this context, exogenous administration of SCFAs delivered via dietary supplementation, in the drinking water, oral gavage, via intraperitoneal (IP) injection, and other routes, has been done in rodents and humans [189,190]. Of note, orally delivered SCFAs reach the gastrointestinal (GI) tract, then signal local SCFAs receptors, rather than being immediately absorbed by colonocytes. On the other hand, IP injection of SCFAs at supra-physiological concentrations rapidly targets organs beyond the gut, thus exerting an acute and potent response on host metabolism [189,190]. Because host metabolic outcomes can be heterogeneous depending on the method of SCFA delivery, interpretation of these study results should be done with care. Various studies that delivered SCFA endogenously to rodents and humans will be described below.

### 6.1. Effects of Exogenously-Administered SCFAs in Rodent Models

Several studies have examined obesity phenotypes in mice given various doses and combinations of SCFA admixed into a HFD. Gao et al. showed that adding butyrate (5% wt/wt) to a HFD increased energy expenditure, thermogenic function, and insulin sensitization and prevented body weight gain in mice [68]. A similar study, in which the HFD was also supplemented with 5% wt/wt SCFA for 12 weeks, showed that acetate, propionate, and butyrate all individually prevented diet-induced obesity and insulin resistance, with similar increases in fatty acid oxidation and energy expenditure [177]. In line with these studies, SCFAs incorporated into an obesogenic diet reduced body weight, mitigated systemic inflammation and other obesity-related markers in mice, with acetate exerting the major effects [133]. An experimental design by Arnoldussen et al. revealed that dietary delivery of butyrate (5%) ameliorated HFD-induced obesity and associated complications, contributing to weight loss and decreased adiposity in mid-adult LDLR KO mice [174]. Thus, there is broad consensus that dietary supplementation of SCFA promotes metabolic benefits that mitigate HFD-induced obesity [68,133,177].

Chronic dietary butyrate supplementation prevented HFD-induced body weight gain and adiposity, likely due to increased fat oxidation and thermogenesis, and improved overall lipid and glucose metabolism in apoE*3-Leiden+CETP, a mouse model with a humanized lipid profile [187]. Additionally, acute oral delivery of butyrate was shown to decrease food intake and neuronal signals, suggesting a connection between the gut–brain axis and energy homeostasis [187]. Further, a single oral gavage of 1.5% acetate increased energy expenditure in parallel with suppression of body weight and fat mass in C57BL/6J mice [173], which was attributed to upregulation of PPARγ and fatty acid oxidation-related genes [175].

Interestingly, propionate added to drinking water or administered via IP injection conferred weight gain, severe hyperinsulinemia, and hyperglycemia as opposed to rectally administered propionate in mice [188]. Such metabolic responses appear to be partially mediated by the adipokine fatty acid-binding protein 4 (FABP4) [188]. Mice fed an obesogenic diet, then treated with IP injections of liposome-encapsulated acetate nanoparticles, showed reduced whole-body adiposity, insulin resistance, and lipolytic activity [181]. Herein, thermogenic function through browning of WAT was increased by these acetate nanoparticles with a coincident reduction in adiposity [181].

Administration of SCFAs similarly impacts lipid metabolism in rats [176,178]. First, intraperitoneal (IP) injections of acetate, given 5 times per week for 6 months, led to decreased body weight while increasing energy expenditure [176]. Acetate also increased lipolytic and lipogenic gene expression in this rat model of spontaneous diabetes [176]. In another study using rats, IP administration of a mixture of SCFAs at a 3:1:1 molar ratio for 7 days had no effects on body weight and abdominal adiposity [178], implying that a longer treatment period is required to exert a body weight phenotype. However, acetate, propionate, and butyrate impacted lipid and glucose metabolism as evidenced by reduced total cholesterol, triglycerides, and glucose levels in a sex-dependent manner [178]. Therefore, SCFAs may exert positive metabolic benefits in both sexes via distinct mechanisms when administered exogenously [178].

Collectively, these discrepant findings could be attributed to variable substrate metabolism or host microbiota signatures, thus leading to diverse physiological and metabolic outcomes in responses to exogenous SCFAs [189,190]. As such, the unique host-specific and site-specific mechanisms whereby SCFAs affect adipose tissue and energy balance regulation should be further investigated. The effects of SCFA administration via different routes on aspects of obesity and related complications in rodents are detailed in Table 4.

### 6.2. Effects of Exogenously Administered-SCFAs in Humans

Mechanistic evidence from rodent studies has shown that the various routes to deliver exogenous SCFAs affect host whole-body substrate and energy metabolism [173,174,176,178,181,187,188]. Yet, the previously described long-term strategies as well as metabolic effects may not all directly translate into humans to prevent and/or manage obesity complications (reviewed extensively in [191]), thus warranting additional investigation.

In one of the first human randomized controlled trials, dietary supplementation of inulin-propionate ester increased the secretion of gut hormones PYY and GLP-1, leading to a reduction in energy intake, body weight, intra-abdominal adipose tissue mass, and plasma cholesterol, HDL, and LDL [192]. Additional evidence demonstrated that orally-delivered propionate modulated energy balance via the promotion of whole-body lipid oxidation in healthy subjects [71], in line with a previous study conducted in mice [177]. Conversely, a small pilot investigating orally-delivered butyrate showed no improvements in glucose metabolism or thermogenic function in insulin-resistant individuals with obesity, reinforcing the concept that butyrate could have been quickly metabolized by colonocytes and may not have been readily available to extra-intestinal organs in a systemic manner [182]. Moreover, butyrate increased total cholesterol and LDL levels after 4 weeks of supplementation compared with healthy lean subjects [182]. In another study, Harstra et al. determined that oral butyrate had no effect on body weight, energy expenditure, and insulin sensitivity in people with MetS, but favored a decrease in metabolic parameters including HbA1c [186]. The lack of effects on body weight with oral butyrate supplementation may be due to lower doses of butyrate relative to body weight as well as a lack of daily dose adjustment, as suggested previously [68,177]. Acetate and acetic acid (vinegar) supplementation has been reported to counteract the deleterious effects of obesity and related complications, hence conferring healthy metabolic functions among host species [193]. Kondo et al. first revealed that continuous oral intake of vinegar lowered weight gain and visceral and subcutaneous fat mass as well as serum triglycerides levels, with no changes in energy balance in a Japanese obese cohort [175]. These results suggest that oral delivery of various SCFA preparations can ameliorate metabolic syndrome complications, thus having a clinical contribution in humans [175].

Notably, distinct routes of SCFA administration markedly impact the metabolic responses in obesity. Rectal or intravenous (IV) infusions with acetate increased gut-derived PYY and GLP-1 secretion, which was accompanied by decreased ghrelin in hyperinsulinemic, overweight woman [185]. In a situation of excessive caloric intake, microbial acetate production was increased, ultimately promoting insulin secretion and whole-body metabolism [193]. Colonic-delivered acetate markedly affected energy expenditure and fat oxidation, then increased circulating glucose, insulin, and PYY in overweight and obese men [183]. Further, supraphysiological rectal infusions of a SCFA mixture for four days modulated energy balance via enhancing fat oxidation and PYY, while attenuating lipolysis [184]. Various studies depicting SCFA administration in humans followed by their metabolic responses are listed in Table 5.

## 7. Gut-Derived SCFAs to Modulate Adipose Tissue Lipolysis

The contributions of SCFAs to host adipose tissue and obesity are becoming increasingly recognized [194,195,196,197,198]. For instance, experimental designs have centered on the mechanisms whereby SCFAs differentially impact signal transduction pathways to regulate lipid and glucose metabolism in WAT, such as lipolysis [138,140,145,165,199,200,201,202,203,204,205]. Briefly, AT lipolysis is a catabolic process leading to the hydrolysis of triacylglycerols (TAGs) into glycerol and free fatty acids (FFA) [206], a process that is initiated when the mobilization of energy stored in adipocytes is required. Hence, targeting adipocyte-specific mechanisms to reduce lipolysis, and increase fat oxidation and energy expenditure, may be crucial for the management of metabolic diseases. In this section, we will summarize the effects of SCFAs on AT lipolysis in vitro and in vivo.

In primary adipocytes from mice and in 3T3-L1 adipocytes, exposure to acetate and propionate (0.1–0.3 mM) inhibited lipolysis and plasma FFA levels in a dose-dependent manner through activation of GPCR-FFAR2 [138]. Treatment with butyrate (≥0.2 mM) and TNF-α attenuated lipolysis as well as glycerol and FFA release in co-cultured 3T3-L1 and RAW264.7 macrophages, which correlated with the decreased protein expression of adipose triglyceride lipase (ATGL), HSL, and phosphorylation of HSL_(Ser660)_ [140]. Under supraphysiological concentrations, Aberdein et al. demonstrated that acetate (4 mM) reduced non-esterified fatty acid (NEFA) and phosphorylation of HSL_(Ser563)_, with no changes in glycerol release, likely via FFAR2 in mature 3T3-L1 adipocytes [202]. Similarly, propionate and butyrate (10 mM) inhibited lipolysis, followed by a reduction in isoproterenol-induced phosphorylation of HSL_(Ser563)_ and HSL_(Ser562)_ in rat and human adipocytes, respectively, an effect that involved FFAR2 [203]. Further, research conducted by Rumberger et al. indicated that butyrate (5 mM) increases the rate of lipolysis in 3T3-L1 adipocytes, underscoring the effects of butyrate on HDAC inhibitory activity [165]. Findings to date showed that SCFA mixtures, in particular those that include acetate, impact lipolysis via attenuation of HSL_(Ser650)_ phosphorylation in human adipocytes [145]. Collectively, these results in cultured cells suggest that SCFAs play an important role in the lipolytic pathway, with the potential to modulate WAT lipid buffering capacity, insulin sensitivity, and improve energy balance.

In in vivo settings, rodents orally given acetate exhibited an increase in the transcription of genes involved with energy metabolism and lipolysis, including long-chain acyl-CoA dehydrogenase (LCAD), 3-ketoacyl-Coa thiolase (3KACT), and PPARγ [176]. Moreover, acetate suppressed lipolysis in subcutaneous AT and decreased circulating FFA levels, mainly through the downregulation of mRNA expression of ATGL as well as target genes involved in fatty acid oxidation and energy expenditure, carnitine palmitoyltransferase I (CPT1) and acyl-CoA oxidase 1 (ACOX1), in HFD-fed mice [181]. Lu et al. subsequently found that long-term dietary supplementation with acetate, propionate, and butyrate promoted a decrease in adipose HSL expression, without affecting CPT1 and FFA levels [133]. In humans, an initial study showed no changes in plasma glycerol, NEFA release, or fat oxidation following the consumption of 2 mg acetate [204]. Conversely, in another study, acetate blunted whole-body lipolysis, as indicated by decreased plasma glycerol, in healthy men [205] and in men with overweight/obesity [184]. In healthy individuals, acute SCFA infusion reduced circulating FFA concentrations [93,199], indicating that lipolysis was reduced [200]. In addition, recent cross-sectional analyses have reported that circulating rather than fecal SCFAs are negatively correlated to systemic FFA and whole-body lipolysis, suggesting that SCFAs impact lipid metabolism, which can improve peripheral insulin sensitivity and ultimately benefit metabolic health in humans [201]. In addition, measuring systemic SCFA levels is more predictive of metabolic status than stool SCFA content [201].

## 8. Conclusions

Over the last several decades, a handful of studies have been performed to highlight gut microbi-derived SCFAs as novel therapeutic targets for managing the risk of obesity-related metabolic disorders. Herein, we have provided an update in this field to show that SCFAs influence adipose tissue biology and physiology and thus impact body weight control, likely via activation and/or inhibition of multiple signaling pathways associated with regulating feeding behavior, ameliorating inflammatory responses, and favorably impacting lipid metabolism. In this contribution, we have described how different host species with varied genetic backgrounds and unique gut microbiota patterns, dietary sources, and composition as well as different methods of exposure to SCFAs could explain the underlying mechanisms whereby SCFAs can directly impact adipose tissue and host metabolic health. Taken together, while the beneficial effects of SCFAs on some adipose tissue functions have been described, the conflicting findings across studies provide a rationale for additional long-term and well-controlled investigations. Furthermore, a complete regulatory system focused on the interplay among host adipose tissue, host genetics, and SCFAs should be explored to inform potential treatment strategies for obesity and chronic diseases.

## Figures and Tables

**Table 2:** Short chain fatty acid receptor signaling in host adipose tissue metabolism in vitro and ex vivo.

Model	Experimental Design	FFAR Expression	Metabolic Response	Reference
3T3-L1 adipocytes	1 mM acetate and propionate for 7 days	↑ FFAR2	↑ adipogenesis, PPARγ	Hong et al., 2005 [139]
		↔ FFAR3	↑ lipid infiltration	
			↓ lipolysis	
	0.1 to 0.3 mM acetate and propionate for 4 h	↑ FFAR2	↓ lipolysis (FFA, glycerol)	Ge et al., 2008 [138]
	10 mM acetate for 2 h	↑ FFAR2	↓ insulin-induced AKT, fatty acid uptake, LPL	Kimura et al., 2013 [132]
	Up to 0.01 mM acetate and propionate for 10 days	↑ FFAR2 ↔ FFAR3	↑ adipogenesis, lipid infiltration ↔ leptin	Frost et al., 2014 [141]
	Up to 1 mM propionate for 30 min	↑ FFAR3	↑ glucose uptake	Han et al., 2014 [143]
Primary murine adipocytes	1 to 3 mM acetate, propionate, and butyrate for 10 min	↑ FFAR3	↑ leptin ↓ cAMP	Xiong et al., 2004 [131]
FFAR3-KO adipocytes	Up to 03 mM acetate, propionate, and butyrate for 4h	↔ FFAR3	↓ leptin ↓ lipolysis	Zaibi et al., 2010 [144]
Human OAT and SAT	Up to 10 mM Propionate for 24 h	↑ FFAR2/3	↑ leptin	Al-Lahham et al., 2010 [150]
			↔ adiponectin	
			↓ inflammation, resistin	
Human OAT	0.01 mM acetate and propionate for 13 days	↔ FFAR2	↔ adipogenesis, aP2 ↓ inflammation	Dewufl et al., 2013 [137]
Human MSCs	3.3 mM propionate for 14 days	↑ FFAR2	↓ adipogenesis, PPARγ ↓ lipid accumulation	Iván et al., 2017 [136]
Human MADs	Up to 1 mM acetate, propionate, butyrate or mixed SCFAs for 6 h	↑ FFAR2/3	↓ lipolysis ↓ phospho-HSL_ser650_	Jocken et al., 2017 [145]

**Table 3:** Gut microbial-derived SCFAs influence adipose tissue inflammation.

Model	Experimental Design	Inflammatory Responses	Reference
Human OAT	3 mM propionate for 24 h	↓ IL-4, IL-10, G-CSF, IP-10, MIP-1α/β, CCL5, TNF-α	Al-Lahham et al., 2012 [147]
THP-1 Mϕ	Up to 10 mM propionate for 2 h	↓ IL-10, G-CSF, MCP-1, CCL5, TNF-α	Al-Lahham et al., 2012 [147]
THP-1 Mϕ	100 mM acetate, 10 mM propionate, and 2 mM butyrate for 24 h	↑ MCP-1 ↓ NF-κB, MAPKs (p38, ERK1/2	Al-Roub et al., 2021 [171]
3T3-L1 and RAW264.7 Mɸ	Up to 1 mM butyrate for 24 h	↓ TNF-α, MCP-1, IL-6 ↓ NF-κB, MAPKs (p38, ERK1/2, JNK1/2)	Ohira et al., 2013 [140]
		↑ PGE2, COX2, cPLA2 ↓ PRKAR1A, cAMP	Ohira et al., 2016 [167]
Mϕ-BMD	Up to 1 mM butyrate between 1.5 to 24 h	↑ Arg1, Fizz1, Ym1	Ji et al., 2016 [170]
		↑ CCL2, CCL17, CCL22	
		↑ H3K9, STAT6	
C57BL6/J mice	5% w/w butyrate for 8 weeks	↑ Mɸ M2 (CD206+)	Kushwaha et al., 2022 [169]
		↑ Tregs (CD25+)	
		↑ IL-10, DioA2, Pgc-1, IL-4	
3T3-L1	1 mM butyrate for 24 h	↓ ER (pPERK, CHOP)	Kushwaha et al., 2022 [169]
Monocytes and ATM	300 mM acetate or 30 mM propionate, and butyrate for 15 h	↓ NF-κB, TNF-α, IL-6	Eslick et al., 2022 [168]
		↓ MAPK1	

**Table 4:** Exogenous administration of SCFAs to impact adipose tissue metabolism in rodents.

Rodent Studies	Experimental Design	Metabolic Response	Reference
C57BL/6J mice	5% wt/wt butyrate via diet supplementation for 16 weeks	↓ body weight	Gao et al., 2009 [68]
		↑ energy expenditure	
		↑ PGC-1α, UCP-1	
		↑ insulin sensitivity	
C57BL/6J mice	5% wt/wt acetate, propionate, butyrate via diet supplementation for 12 weeks	↓ body weight, WAT mass, adipocytes size	den Besten et al., 2015 [177]
		↑ energy expenditure, fat oxidation,	
		↑ insulin sensitivity	
C57BL/6J mice	5% wt/wt^−1^ acetate, propionate, and butyrate or SCFAs mixture diet supplementation for 16 weeks	↔ food and energy intake	Lu et al., 2016 [133]
		↓ body weight	
		↓ glucose, FFA, IL-1β, IL-6, MCP-1	
LDLR KO-Leiden mice	5% wt/wt butyrate via diet supplementation for 15 weeks	↔ food and energy intake	Arnoldussen et al., 2017 [174]
		↓ body weight, omental and inguinal fat	
		↓ cholesterol, triglycerides, insulin	
ApoE*3-Leiden.CETP	5% wt/wt butyrate via diet supplementation for 9 weeks 6 M butyrate via oral gavage/15 or 150 mM IV injection	↓ food intake	Li et al., 2018 [187]
		↓ body weight, epididymal WAT	
		↓ plasma triglycerides, insulin	
		↑ fat oxidation and BAT activity	
C57BL/6J mice	1.5% acetate via oral gavage for 1 day	↑ energy expenditure	Hattori et al., 2010 [173]
		↓ body weight and fat mass	
C57BL/6J mice	(1) ~15 mg/kg propionate via drinking water for 6 weeks	↑ body weight	Tiroshi et al., 2019 [188]
	(2) I.P at 0.5 to 2 g/kg of BW	↑ glucose, insulin	
	(3) 1g/kg of BW rectal infusion	↑ FABP4	
C57BL6/J mice	Liposome encapsulated acetate nanoparticle via I.P. at 1g/kg of BW for 6 weeks	↔ body weight, adipocytes	Sahuri-Arisoylu et al., 2016 [181]
		↓ whole-body adiposity, lipolysis, FFA	
		↑ insulin sensitivity	
		↑ browning (UCP1, PRDM16)	
Otsuka Long-Evans Tokushima Fatty rats	acetate via I.P. at 5.2 mg/kg of BW for 6 months	↓ body weight, lipid droplets size	Yamashita et al., 2009 [176]
		↑ energy balance	
		↑ LCAD, 3KACT, PPARγ	
Long-Evans rats	acetate, propionate, butyrate via I.P at 60:20:20 M of BW for 7 days	↔ body weight, abdominal fat and food intake, NEFA	Shah et al., 2021 [178]
		↓ cholesterol, triglycerides, glucose	

**Table 5:** Routes of administration of SCFAs to impact adipose tissue metabolism in humans.

Human Studies	Experimental Design	Metabolic Response	Reference
Healthy men/women	10 g/day inulin-propionate via dietary supplementation for 24 weeks	↓ food intake, body weight and intra-abdominal AT	Chambers et al., 2015 [192]
		↑ PYY, GLP-1	
		↓ cholesterol, HDL, LDL	
	6.8 g propionate via oral administration for 2 h/2 days	↑ energy expenditure	Chambers et al., 2018 [71]
		↑ lipid oxidation	
Healthy and MetS male	4 g/day butyrate via oral administration for 4 weeks	↔ BMI, BAT activity	Bouter et al., 2018 [182]
		↔ energy expenditure, insulin sensitivity	
		↑ cholesterol, LDL	
MetS males/females	4 g/day via oral administration butyrate for 4 weeks	↔ body weight,	Harstra et al., 2020 [186]
		↔ energy expenditure, insulin sensitivity	
		↓ Cholesterol, triglycerides, HbA1c	
Men/women with obesity	Up to 1.5 g acetate via oral administration for 12 weeks	↔ energy expenditure	Kondo et al., 2009 [175]
		↓ body weight	
		↓ visceral, subcutaneous fat mass	
		↓ triglycerides	
Hyperinsulinemic and overweight women	60 mM/L acetate via rectal infusions and 20 mM/L via IV for up to 1 h	↔ glucose and insulin	Freeland et al., 2010 [185]
		↑ PYY, GLP-1	
		↓ ghrelin	
Men with overweight and/or obesity	Up to180 mM/L acetate via rectal infusions for 3 days	↑ energy expenditure, fat oxidation	Van der Beek et al., 2016 [183]
		↑ glucose, insulin, PYY	
	Up to 200 mM/L SCFAs mixture via rectal infusions for 4 days	↑ energy expenditure, fat oxidation	Canfora et al., 2017 [184]
		↑ PYY	
		↓ lipolysis	

## Abbreviations

10,12 CLA: trans-10,cis-12 conjugated linoleic acid; 3KACT: 3-ketoacyl-CoA thiolase; ACOX1: acyl-CoA oxidase 1; AKT: protein kinase B; AMPK: AMP-activated protein kinase; aP2: adipocyte fatty acid-binding protein 2; apoE. Apolipoprotein E; Arg1: arginase 1; AT: adipose tissue; ATGL: adipose triglyceride lipase; ATM: adipose tissue macrophage; BAT: brown adipose tissue; BMI: body mass index; C2: acetate; C3: propionate; C4: butyrate; CAD: coronary artery disease; cAMP: cyclic adenosine monophosphate; CCL17: CC motif chemokine ligand 17; CCL2: CC motif chemokine ligand 2; CCL22: CC motif chemokine ligand 22; CCL5: CC motif chemokine ligand 5; CEBPα: CCAAT/enhancer-binding protein alpha; CHOP: CEBP homologous protein; CETP: cholesteryl ester transfer protein; COX2: cyclooxygenase-2; cPLA2: cytosolic phospholipase A2; CPT1: carnitine palmitoyltransferase I; DioA2: iodothyronine deiodinase-2; ER: endoplasmic reticulum; ERK1/2: extracellular signal-regulated protein kinase 1/2; FABP4: fatty acid binding protein 4; FABP4: fatty acid-binding protein 4; FFA: free fatty acid; FFAR2: free fatty acid receptor 2 (GPR43); FFAR3: free fatty acid receptor 3 (GPR41); Fizz1. Found in inflammatory zone protein 1; GF: germ free; GI: gastrointestinal; GLP-1: glucagon-like peptide 1 (GLP-1); GPCR: G-protein coupled receptor; G-CSF: granulocyte colony-stimulating factor; H3K9: histone H3 lysine 9; HCA2: hydroxycarboxylic acid receptor; HDAC: histone deacetylase; HFD: high fat diet; HFHS: high fat high sucrose; hMADS: human multipotent adipose tissue-derived stem cells; HSL: hormone sensitive lipase; IL-1ꞵ: interleukin 1ꞵ; IL-4: interleukin 4; IL-6: interleukin 6; IP: intraperitoneal; IP-10: interferon-gamma induced protein; IV: intravenous; JNK 1/2: c-Jun N-terminal kinase; LCAD: long-chain acyl-coenzyme A (acyl-CoA) dehydrogenase; LDLR: low-density lipoprotein receptor; LPL: lipoprotein lipase; LPS: lipopolysaccharide; Mɸ: macrophage; M1: classically activated macrophage; M2: alternatively activated macrophage; MAPK: mitogen-activated protein kinases; MCP-1: monocyte chemoattractant protein—1; MetS: metabolic syndrome; MIP-1α/ꞵ: macrophage inflammatory proteins-1 alpha and beta; MKK3: mitogen-activated protein kinase 3; MMe: metabolically activated macrophage; mRNA: messenger RNA; MSC: mesenchymal stem cells; NEFA: non-esterified fatty acids; NF-ᴋB: nuclear factor kappa B; OAT: omental adipose tissue; OLFR78: olfactory receptor 78; Pgc-1: peroxisome proliferator-activated receptor gamma coactivator 1 alpha; PGE2: prostaglandin E2; PPARγ: peroxisome proliferator-activated receptor gamma; pPERK: phospho-protein kinase-like ER kinase; PRAK: p38-regulated activated protein kinase; PRDM16: PR-domain containing 16; PRKAR1A: c-AMP-dependent protein kinase type 1-alpha; PYY: peptide YY; RER: respiratory exchange ratio; RNA: ribonucleic acid; ROS: reactive oxygen species; SAT: subcutaneous adipose tissue; SCFA: short chain fatty acid; siRNA: small interfering RNA; STAT6: signal transducer and activator of transcription 6; T2D: type 2 diabetes; TNF-α: tumor necrosis factor alpha; UCP1: uncoupling protein 1; WAT: white adipose tissue; Ym1: chitinase-like protein.
